# Enabling safe aqueous lithium ion open batteries by suppressing oxygen reduction reaction

**DOI:** 10.1038/s41467-020-16460-w

**Published:** 2020-05-26

**Authors:** Long Chen, Longsheng Cao, Xiao Ji, Singyuk Hou, Qin Li, Ji Chen, Chongyin Yang, Nico Eidson, Chunsheng Wang

**Affiliations:** 1 Department of Chemical and Biomolecular Engineering, University of Maryland, College Park, MD 20742 USA; 2 Department of Chemistry and Biochemistry, University of Maryland, College Park, MD 20742 USA

**Keywords:** Electrochemistry, Batteries, Energy

## Abstract

Due to the non-flammable nature of water-based electrolytes, aqueous lithium-ion batteries are resistant to catching fire. However, they are not immune to the risk of explosion, since the sealing structure adopted by current batteries limits the dissipation of heat and pressure within the cells. Here, we report a safe aqueous lithium-ion battery with an open configuration using water-in-salt electrolytes and aluminum oxide coated anodes. The design can inhibit the self-discharge by substantially suppressing the oxygen reduction reaction on lithiated anodes and enable good cycle performance over 1000 times. Our study may open a pathway towards safer lithium-ion battery designs.

## Abstract (web summary)

Despite the non-flammable nature of water-based electrolytes, aqueous lithium-ion batteries still carry an explosion risk due to the sealing structure. Here the authors report a safe aqueous battery with an open configuration, utilizing highly concentrated electrolytes and Al_2_O_3_ coated anodes.

## Introduction

**Fig. 1:**
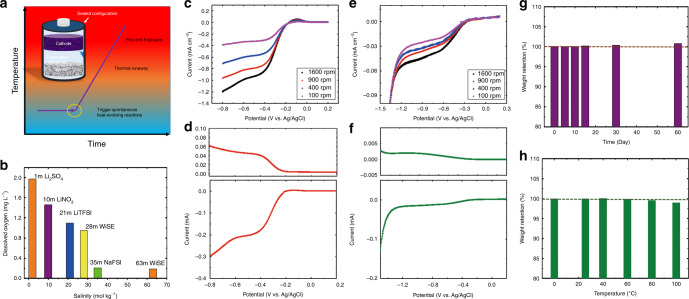
The safety of commercial LIB and the oxygen reduction reaction (ORR) in the “water-in-salt electrolytes” (WiSE) and the “salt-in-water electrolytes” (SiWE). **a** Typical thermal runaway in a Li-ion cell with sealed-configuration. **b** Solubility of O_2_ in aqueous electrolytes. **c** Linear sweep voltammetry (LSV) of carbon black in the O_2_-saturated 1 m SiWE at a scan rate of 10 mV s^−1^ with different rotating disk electrode (RDE) rotation rates. **d** LSV of carbon black in the O_2_-saturated 1 m SiWE and the corresponding ring current for the oxidation of hydrogen peroxide at a scan rate of 10 mV s^−1^ with a disc surface area of 0.2475 cm^2^, a rotating ring disk electrode (RRDE) collection efficiency of 0.37, and an electrode rotation rate of 1600 rpm. **e** LSV of carbon black in the O_2_-saturated 28 m WiSE at a scan rate of 10 mV s^−1^ with different RDE rotation rates. **f** LSV of carbon black in the O_2_-saturated 28 m WiSE and the corresponding ring current for the oxidation of peroxide at a scan rate of 10 mV s^−1^ with a disc surface area of 0.2475 cm^2^, an RRDE collection efficiency of 0.37, and an electrode rotation rate of 1600 rpm. **g** The mass retention of the 28 m WiSE in the air at room temperature with a relative humidity of ~68%. **h** The mass retention of the 28 m WiSE at different temperatures for 30 min.

The safety of lithium-ion batteries (LIBs) has raised significant concerns in recent years due to several fire-related incidents^1–3^. The fully charged LIB consists of a highly energetic transition metal oxide cathode and a lithiated graphite anode in intimate contact with a flammable organic electrolyte. Any abuse by overcharging, external short-circuiting, or crushing can trigger spontaneous heat-generation. This exothermic event can result in thermal runaway, or even explosion due to high internal cell pressure producing fire and toxic gases. The thermal stability of batteries depends on the electrolyte’s flammability and its ability to dissipate the heat and pressure. Currently, all commercial LIBs are in sealed configurations to protect the highly reactive electrodes and liquid electrolytes from reacting with the moisture and the reactive gases in the air, inherently limiting the dissipation of heat and pressure (Fig. 1a). Although many safety devices, such as safety vents, thermal fuses, circuit breakers, positive temperature coefficient elements, and shutdown separators, have been applied to LIBs and battery packs^3^, the fire and explosion risks of LIBs are still present. To make LIBs intrinsically safe, the LIBs’ chemistry and configuration have to be changed.

At the chemistry level, the volatile, flammable, and toxic organic electrolytes in commercial LIBs should be replaced by non-flammable aqueous electrolytes^4–8^. However, the traditional non-flammable aqueous LIBs suffer from a low energy density due to the narrow electrochemical stability window of aqueous electrolytes^9^. The performance of aqueous LIBs is significantly impacted by the dissolved O_2_ in electrolytes from the air through the oxygen reduction reaction (ORR). Xia and coworkers^10^ have demonstrated that any discharged negative electrode in an aqueous LIB would react with O_2_, resulting in the marked capacity fading. Sealing cell can help to eliminate the O_2_ and can significantly improve the battery performance. But sealed aqueous LIBs still have the risk of thermal runaway and explosion when the internal cell pressure quickly rises, either by electrochemical water decomposition or uncontrolled high-temperature thermo-vaporization, as observed in sealed aqueous Ni-MH and VRLA (valve regulated lead acid) batteries. The battery’s vents are not always able to prevent cell explosions because the pressure builds up from accelerated side reactions producing gas at a rate too fast for the safe operation of the safety valves, leading to the dangerous cell explosions. The sealed aqueous LIBs have to be replaced by an open configuration design due to their superior ability to dissipate heat and pressure. To our best knowledge, such open configuration aqueous LIBs have not been demonstrated yet. Recently, we developed a “water-in-salt electrolyte” (WiSE) that significantly reduced the O_2_ solubility in the electrolyte and expanded the electrochemical stability window of aqueous electrolytes from 1.5 to >3.0 V. The WiSE enabled the energy density of aqueous LIBs to be significantly enhanced from 75 Wh kg^−1^ to over 300 Wh kg^−1^ (on the material level)^11–16^. The uniqueness of WiSE has been extensively reviewed by Eftekhari^9^.

Here in this study, we report intrinsically safe LIBs with an open configuration. It is found that the O_2_ solubility markedly decreases in the electrolytes from 1.97 mg L^−1^ in 1 m “salt-in-water electrolytes” (SiWE) (1 m Li_2_SO_4_) to 0.95 mg L^−1^ in 28 m WiSE (21 m LiTFSI + 8 m LiOTf, mol per kilogram), and further reduces to 0.19 mg L^−1^ in 63 m WiSE, significantly suppressing the ORR on lithiated anodes. The ORR on lithiated anodes can be further inhibited by coating 2.0-nm-thick aluminum oxide (Al_2_O_3_) layer on anodes. Furthermore, the 28 m WiSE can prevent water evaporation even at a high temperature of ∼100 °C due to the strong hydrophilic property of the LiTFSI salt. The LiMn_2_O_4_//Al_2_O_3_@LiTi_2_(PO_4_)_3_ open power cell with 28 m WiSE demonstrates an energy density of 62.4 Wh kg^−1^ and a stable cycle life of >1000 cycles. The 0.1 Ah LiVPO_4_F//Al_2_O_3_@Li_4_Ti_5_O_12_ open pouch cell with 63 m WiSE (42 m LiTFSI + 21 m Pyr_13_·TFSI) yield an energy density of 170 Wh kg^−1^ and a high energy efficiency of 92.8% for over 50 cycles.

## Results

### WiSE enabled open configuration

**Fig. 2:**
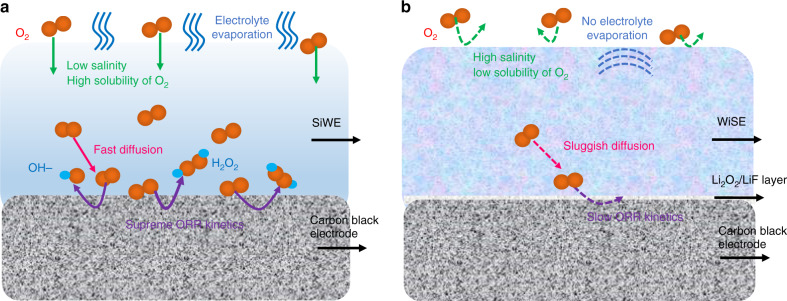
Schematic of the ORR at the carbon black electrodes in the SiWE and the WiSE. **a** Schematic of the ORR in the 1 m SiWE. **b** Schematic of the ORR in the 28 m WiSE.

The low O_2_ content in the 28 m WiSE reduces the ORR kinetics at the electrodes. In order to build an open aqueous battery, the ORR kinetics at the electrodes must be reduced. The gas solubility in a solvent can be largely reduced by increasing the salt concentration in the solvent^17^. Figure 1b shows that the solubility of O_2_ in the water markedly decreases from 1.97 mg L^−1^ in the 1 m SiWE to 0.95 mg L^−1^ in the 28 m WiSE, further down to 0.21 mg L^−1^ in the 35 m WiSE (35 m NaFSI in H_2_O), and even further to 0.19 mg L^−1^ in the 63 m WiSE. When an ionic salt is added to water, the dissolved ions will attract the water molecules due to ion solvation. The solvation of ions by water will reduce the affinity of dissolved non-polar O_2_ to water, thus reducing the solubility of O_2_ in water. The low O_2_ content in electrolytes reduces the ORR kinetics at the electrodes. Using carbon black as a model electrode (the most reactive component in conventional anodes), the ORR performances of carbon black in the O_2_-saturated 28 m WiSE and in the O_2_-saturated 1 m SiWE were investigated using the RDE technique. The ORR kinetics on carbon black were significantly reduced with the increase of salt concentration in O_2_-saturated aqueous electrolytes, as demonstrated by three pieces of evidence in RDE measurements at 1600 rpm (Fig. 1c, e): (1) The onset of the ORR potential at carbon black electrodes, where the reduction current increases sharply, (−0.3 V vs. Ag/AgCl) in the 28 m WiSE (Fig. 1e) is more negative than the onset ORR potential (−0.2 V vs. Ag/AgCl) in the 1 m SiWE (Fig. 1c); (2) The limiting current of carbon black electrodes in the 28 m WiSE (0.045 mA cm^−2^) is only 4.5% of the limiting current (1.0 mA cm^−2^) in the 1 m SiWE; and (3) The half-wave potential (−0.45 V vs. Ag/AgCl) of the ORR in the 28 m WiSE is much more negative than that (−0.28 V vs. Ag/AgCl) in the 1 m SiWE. The much lower reduction current of the carbon black electrodes in the N_2_ saturated 28 m WiSE and 1 m SiWE (Supplementary Fig. 1) than those in the O_2_ saturated electrolytes at the same conditions (Fig. 1c, e) confirmed that the currents in Fig. 1c, e are attributed to the ORR.

The low diffusion coefficient in the 28 m WiSE further reduces the ORR kinetics on the electrodes. In addition to decreasing the O_2_ solubility, the high salt concentration also reduces the diffusion coefficients of O_2_ in the electrolytes. The diffusion coefficients for O_2_ in the 28 m WiSE and the 1 m SiWE were calculated using the Koutecky–Levich (K–L) equation (Eq. 1).

1 $$\frac{1}{{|j|}} = \frac{1}{{|j_{\mathrm{k}}|}} + \frac{1}{{|j_{\mathrm{d}}|}} = \frac{1}{{|j_{\mathrm{k}}|}} + \frac{1}{{0.62\,{\mathrm{nF}}\,D_{{{\mathrm{O}}}_2}^{2/3}C_{{{\mathrm{O}}}_2}\,\nu ^{ - 1/6}\omega ^{1/2}}}$$

Where *j* is the measured current density; *j*_k_ is the kinetic current density; *F* is the Faraday constant (96485 C mol^−1^); $D_{{{\mathrm{O}}}_2}$ is the O_2_ diffusion coefficient in the electrolytes; $C_{{{\mathrm{O}}}_2}$ is the O_2_ saturation concentration in the electrolytes (6.156 × 10^−5^ mol L^−1^ for the 1 m SiWE and 2.968 × 10^−5^ mol L^−1^ for the 28 m WiSE) (Fig. 1b); *ν* is the kinematic viscosity of the electrolyte that is obtained using the viscometer (1.396 mPa s^−1^ for the 1 m SiWE and 202.44 mPa s^−1^ for the 28 m WiSE); *ω* is the electrode rotation rate; and *n* is the electron transfer number (for the 1 m SiWE, *n* can be obtained via the RRDE test using Eq. 2).

2 $${{n}} = 4 - \frac{{4j_{\mathrm{r}}}}{{{{N}}|j_{\mathrm{d}}| + j_{\mathrm{r}}}}$$

Where *j*_r_ and *j*_d_ are the ring and the disk current, respectively, and *N* is the collection efficiency (0.37). From the disk currents of the RRDE in Fig. 1d, the electron transfer number (*n*) in the ORR in the O_2_-saturated 1 m SiWE was determined to be 2.4–2.6 (Supplementary Fig. 2a) using Eq. 2. This value corresponds to 71–80% H_2_O_2_ (Supplementary Fig. 2b, calculated using Supplementary Eq. 1 in Supplementary Note 1) and 29–20% OH^−^ as the ORR products. Hence, the diffusion coefficient of O_2_ ($D_{{{\mathrm{O}}}_2}$) in the O_2_-saturated 1 m SiWE was determined to be 5.45 × 10^−5^ cm^2^ s^−1^. In addition, the ORR products (both H_2_O_2_ and OH^−^) produced at carbon electrode surface quickly dissolve into the 1 m SiWE and diffuse away from the electrode due to their high solubility and diffusivity, further accelerating the ORR kinetics^18^.

In contrast, the main ORR product on the carbon electrode in the O_2_-saturated 28 m WiSE is Li_2_O_2_, which has been confirmed by the two clear Li_2_O_2_ peaks in the Raman spectrum of the discharged carbon electrode (Supplementary Fig. 3, Supplementary Note 2). It is consistent with previous reports in other WiSE^19^. However, limited H_2_O_2_ can still be monitored in the O_2_-saturated 28 m WiSE (Fig. 1f), suggesting that the H_2_O_2_ is possibly an intermediate in the ORR. The possible ORR pathway at the carbon electrode in the 28 m WiSE can be best expressed as follows:

3 $${\mathrm{O}}_2 + {\mathrm{H}}_2{\mathrm{O}} + 2{{\mathrm{e}}}^ - \rightleftharpoons {\mathrm{HO}}_2^ - + {\mathrm{OH}}^ -$$

4 $${\mathrm{HO}}_2^ - + {\mathrm{H}}_2{\mathrm{O}} \rightleftharpoons {\mathrm{H}}_2{\mathrm{O}}_2 + {\mathrm{OH}}^ - $$

5 $${\mathrm{H}}_2{\mathrm{O}}_2 + 2{\mathrm{OH}}^ - + 2{\mathrm{Li}}^ + \rightleftharpoons {\mathrm{Li}}_2{\mathrm{O}}_2 + 2{\mathrm{H}}_2{\mathrm{O}}$$

The reaction mechanism suggests that *n* for the ORR at the carbon electrode in the 28 m WiSE should be 2. The $D_{{{\mathrm{O}}}_2}$ in the 28 m WiSE was determined from the K–L equation (Eq. 1) and the RDE data in Fig. 1e to be 1.54 × 10^−5^ cm^2^ s^−1^. The $D_{{{\mathrm{O}}}_2}$ in the 28 m WiSE is less than the value (5.45 × 10^−5^ cm^2^ s^−1^) in the 1 m SiWE, which further reduces the ORR kinetics at the carbon black electrode in the 28 m WiSE.

The formation of an insulating Li_2_O_2_ and LiF nano passivation layer on the anodes further reduces the ORR kinetics. Finally, the highly insulating Li_2_O_2_ at the carbon electrode does not dissolve in the 28 m WiSE but forms a nano passivation layer, which inhibits the ORR^20–23^. Moreover, an LiF-based solid-electrolyte-interphase (SEI) can also be formed on the anode from reduction of the LiTFSI in the WiSE at around −0.5 to −0.3 V (vs. Ag/AgCl)^5^, further suppressing the ORR.

In addition to the capability of WiSE to reduce the ORR kinetics, it also prevents the water from evaporating. Fig. 1g demonstrates the mass retention of the 28 m WiSE during exposure to the air with a relative humidity of ~68% at room temperature. The 28 m WiSE retains the water content for over 60 days with only a slight mass intake of 0.8%, indicating that the electrolyte actually absorbs moisture from the ambient air. Moreover, even when the 28 m WiSE is exposed to the air at a high temperature of 100 °C (boiling point of water) for 30 min, the mass retention of this electrolyte is still over 99% (Fig. 1h), which indicates that only trace water will evaporate at this high operating temperature. The high stability of the TFSI anion in the aqueous electrolyte at different pH values for 100 days at various temperatures has been shown in a previous study^24^. The 28 m WiSE is stable in the open-cell configuration within a wide temperature window for a long period of time. In summary, the ORR kinetics at the carbon black electrodes in the 1 m SiWE and the 28 m WiSE are schematically shown in Fig. 2. The ORR at the carbon black electrode in the 28 m WiSE is significantly reduced due to the low solubility of O_2_, sluggish O_2_ diffusion, and the formation of a Li_2_O_2_ and LiF nano passivation layer on the electrode. In addition, the high stability of the 28 m WiSE in the air also enables the 28 m WiSE-based cells to be fabricated in an open configuration.

### Al_2_O_3_ suppressing the ORR at the anode surface

The LiF–Li_2_O_2_ nano passivation layer on the anode displays self-healing capability in the 28 m WiSE and can suppress the ORR at the anode surface, thus reducing the cell self-discharge rate and extending the cell cycle life. However, the formation of this passivation layer on the anode consumes the salts and the Li-ions from the lithiated cathode. To suppress the ORR during the initial passivation layer formation process, an Al_2_O_3_ nano-layer was coated on the electrode using ALD to generate an artificial SEI layer prior to cell construction.

**Fig. 3:**
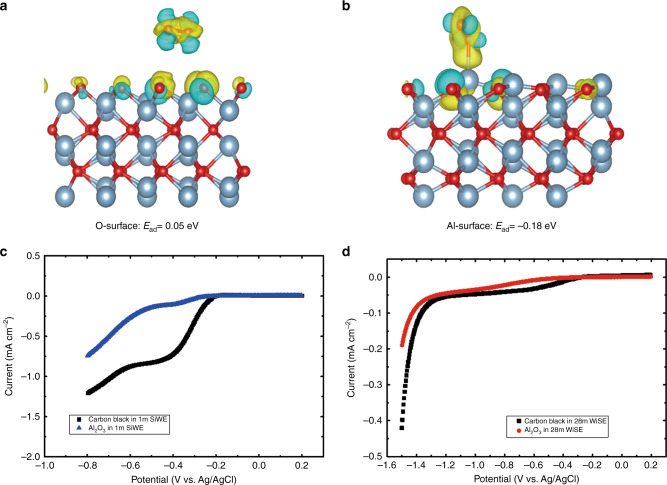
The effect of the Al_2_O_3_ coating on the ORR at the electrode. Different charge densities of O_2_ adsorbed on the O-surface (**a**) and Al-surface (**b**) of Al_2_O_3_. Red and blue spheres represent O and Al atoms, respectively, the corresponding adsorption energies (*E*_ad_) are listed below. **c** LSV of the carbon black and Al_2_O_3_ in the O_2_-saturated 1 m SiWE at a scan rate of 10 mV s^−1^ with a rotation rate of 1600 rpm; **d** LSV of the carbon black and Al_2_O_3_ in the O_2_-saturated 28 m WiSE at a scan rate of 10 mV s^−1^ with a rotation rate of 1600 rpm. The carbon black and Al_2_O_3_ loading on the RDE are 20 μg.

The O_2_ adsorption and the subsequent ORR on the Al_2_O_3_ surface were investigated. Figure 3a, b delivers the different charge densities of the O_2_ adsorbed on the Al_2_O_3_ slab. The corresponding adsorption energies (*E*_ad_) were calculated using Eq. 6:

6 $${{E}}_{{\mathrm{ad}}} = E_{(\mathrm{Al}_{2}{\mathrm{O}}_{3}+{\mathrm{O}}_{2})} - E_{(\mathrm{Al}_{2}{\mathrm{O}}_{3})} - E_{(\mathrm{O}_{2})}$$

Where the ${E}_{({\mathrm{Al}}_{2}{\mathrm{O}}_{3}+{\mathrm{O}}_{2})}$ is the energy of O_2_ adsorbed by the Al_2_O_3_ slab; $E_{({\mathrm{Al}}_{2}{\mathrm{O}}_{3})}$ and $E_{({\mathrm{O}}_{2})}$ are the energy of the Al_2_O_3_ slab and the O_2_, respectively. The more negative adsorption energy indicates the stronger O_2_ adsorption. The typical adsorption energies of the O_2_ intermediates on Pt, Ni, Pd, Cu, and Ir(111) metals surface are −0.72, −1.67, −1.01, −0.56, and −1.27 eV, respectively^25,26^. The binding energy of O_2_ on the O^−^ and the Al-surface of Al_2_O_3_ are around 0.05 and −0.18 eV, demonstrating the weak interaction between the O_2_ and the Al_2_O_3_ surface and the difficulty in the dissociation of the O_2_ on the Al_2_O_3_ surface.

The RDE curves in Fig. 3c, d confirm that the ORR kinetics at the Al_2_O_3_ surface are much slower than the ORR kinetics at the carbon back electrode. Figure 3c shows the LSV of the carbon black and the Al_2_O_3_ in the O_2_-saturated 1 m SiWE, the ORR onset potential of the ORR at the Al_2_O_3_ surface is 0.1 V lower than at the carbon black surface. The limiting current density at 1600 rpm at the Al_2_O_3_ surface (0.107 mA cm^−2^) is only 1/8 of that (0.85 mA cm^−2^) at the carbon black surface. Similarly, the Al_2_O_3_ also reduces the ORR current in the 28 m WiSE (Fig. 3d). The onset potential of the ORR at the Al_2_O_3_ surface is also 0.2 V lower than that at the carbon black surface in the 28 m WiSE (Fig. 3d). In addition, the Al_2_O_3_ has a fast Li-ion diffusivity^27^ and the ALD-Al_2_O_3_ coating technology has been widely used in industry.

### WiSE and Al_2_O_3_ enabling low self-discharge anodes

**Fig. 4:**
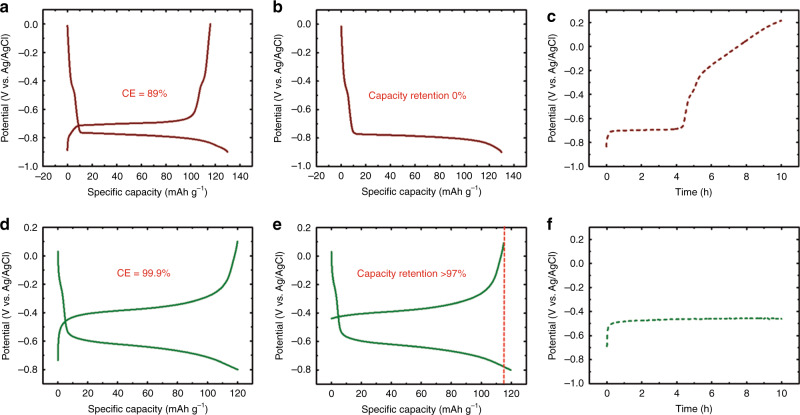
Effects of the ORR on the self-discharge of the lithiated Li_3_Ti_2_(PO_4_)_3_ electrodes in a three-electrodes open-cell configuration. **a** the charge/discharge curves of the LiTi_2_(PO_4_)_3_ electrode in the 1 m SiWE at the current density of 0.5 A g^−1^. **b** The lithiation potential profile of the LiTi_2_(PO_4_)_3_ electrode in the 1 m SiWE at the current density of 0.5 A g^−1^ prior to self-discharge evaluation. **c** The open-circuit potential curve of the discharged LiTi_2_(PO_4_)_3_ in the 1 m SiWE over 10 h of relaxation at open-circuit. **d** The charge/discharge curves of the Al_2_O_3_@LiTi_2_(PO_4_)_3_ electrode in the 28 m WiSE at the current density of 0.5 A g^−1^. **e** The lithiation potential profile and the subsequent delithiation profile of the Al_2_O_3_@LiTi_2_(PO_4_)_3_ electrode at the current density of 0.5 A g^−1^ in the 28 m WiSE after 10 h relaxation at open-circuit. **f** The open-circuit potential profile of the lithiated Al_2_O_3_@Li_3_Ti_2_(PO_4_)_3_ electrode in the 28 m WiSE over 10 h of relaxation at open-circuit. These tests were conducted in an open-cell configuration with exposure to the ambient air.

After assessing the ORR kinetics in the presence of WiSE and Al_2_O_3_ coating, further measurements with a model anode are needed to confirm the above results. As the lithiation/delithiation potential of LiTi_2_(PO_4_)_3_ anode is within the electrochemical stability windows of both the 1 m SiWE and the 28 m WiSE, the LiTi_2_(PO_4_)_3_ anode was used as a model anode to compare the self-discharge behaviors in the 1 m SiWE and the 28 m WiSE. The Coulombic efficiency of the LiTi_2_(PO_4_)_3_ in the 1 m SiWE at the current density of 0.5 A g^−1^ is only 89% (Fig. 4a) and no delithiation capacity is available for the fully lithiated Li_3_Ti_2_(PO_4_)_3_ after 10 h of relaxation at open-circuit in an open-cell (Fig. 4b). The open-circuit potential changed from the fully lithiated potential of −0.7 V to the fully dilithiated potential of +0.2 V during 10 h (Fig. 4c). Therefore, the fully lithiated Li_3_Ti_2_(PO_4_)_3_ in the 1 m SiWE has completely self-discharged to be LiTi_2_(PO_4_)_3_ after 10 h rest in an open-cell configuration. Supplementary Fig. 4 and Supplementary Note 3 confirm the ORR at the LiTi_2_(PO_4_)_3_ electrode. Supplementary Fig. 5 and Supplementary Note 4 further confirm the effects of the dissolved O_2_ in the electrolytes on the self-discharge performance of the lithiated Li_3_Ti_2_(PO_4_)_3_, the open-circuit potential changed to +0.2 V after only a half hour in a pure O_2_ atmosphere, while its lithiated potential can be maintained for 50 h without potential increase when the cell was in the absence of O_2_ (in a N_2_ atmosphere). Therefore, it can be concluded that the self-discharge of the fully lithiated Li_3_Ti_2_(PO_4_)_3_ in the 1 m SiWE open-cell is proceeds through the following ORR pathway:

7 $${\mathrm{O}}_2 + 4{{\mathrm{e}}}^ - + 2{\mathrm{H}}_2{\mathrm{O}} \rightleftharpoons 4{\mathrm{OH}}^ -$$

8 $${\mathrm{O}}_2 + 2{{\mathrm{e}}}^ - + 2{\mathrm{H}}_2{\mathrm{O}} \rightleftharpoons {\mathrm{H}}_2{\mathrm{O}}_2 + 2{\mathrm{OH}}^ -$$

9 $${\mathrm{Li}}_3{\mathrm{Ti}}_2\left( {{\mathrm{PO}}_4} \right)_3 \rightleftharpoons {\mathrm{LiTi}}_2\left( {{\mathrm{PO}}_4} \right)_3 + 2{\mathrm{Li}}^ + + 2{{\mathrm{e}}}^ -$$

In an open-cell configuration, the dissolved O_2_ in the electrolyte receives electrons from the Li_3_Ti_2_(PO_4_)_3_ and is reduced into OH^−^ or H_2_O_2_. As expected, when the Al_2_O_3_ nano-layer coated LiTi_2_(PO_4_)_3_ electrode (Supplementary Figs. 6 and 7) is charged/discharged in the 28 m WiSE, the Coulombic efficiency markedly increases to 99.9% (Fig. 4d). The capacity retention of the fully lithiated Li_3_Ti_2_(PO_4_)_3_ after 10 h rest in an open-cell configuration still reaches a high value of >97% (Fig. 4e). Also, the open-circuit potential is maintained at −0.5 V (Fig. 4f) due to the ORR on the lithiated Al_2_O_3_@Li_3_Ti_2_(PO_4_)_3_ being significantly inhibited. To further confirm the high ORR suppression capability of the WiSE, a moderate salinity concentration electrolyte of 10 m LiTFSI and a high concentration of 30 m WiSE (28 m ZnCl_2_ + 2 m LiCl) were also selected to evaluate the self-discharge of the lithiated Al_2_O_3_@Li_3_Ti_2_(PO_4_)_3_ anodes. As shown in Supplementary Fig. 8 and Supplementary Note 5, only 52% of the discharge capacity remains after 10 h in the 10 m LiTFSI electrolyte, whereas in the 30 m WiSE, the discharged electrode retains over 98% of the initial capacity after a 10 h rest and it maintains over 96% of the initial capacity after 24 h in an open-cell configuration (Supplementary Figs. 9 and 10).

### Electrochemical performance of safe WiSE full open cells

**Fig. 5:**
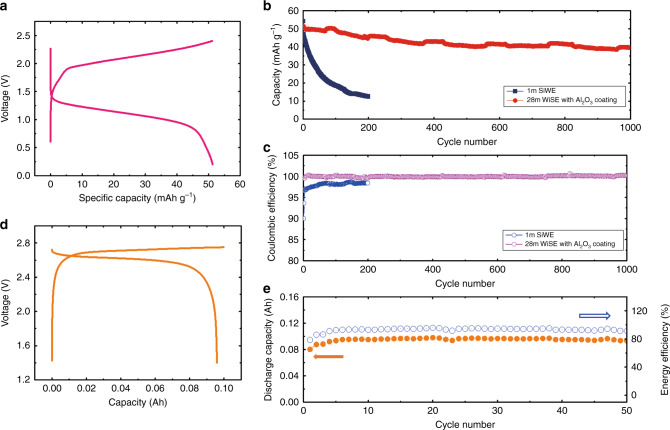
Electrochemical performance of the WiSE full open cells. **a** The charge/discharge curves of the LiMn_2_O_4_//Al_2_O_3_@LiTi_2_(PO_4_)_3_ open-cell with the 28 m WiSE at a rate of 5 C. **b** The cycling performance of the LiMn_2_O_4_//Al_2_O_3_@LiTi_2_(PO_4_)_3_ open-cell with the 28 m WiSE at a rate of 5 C. **c** The Coulombic efficiency of the LiMn_2_O_4_//Al_2_O_3_@LiTi_2_(PO_4_)_3_ open-cell with the 28 m WiSE at a rate of 5 C. **d** The charge/discharge curves of the demo LiVPO_4_F//Al_2_O_3_@Li_4_Ti_5_O_12_ open pouch cell with the 63 m WiSE and a constant charge capacity of 0.1 Ah at a rate of 0.2 C. **e** The cycling performance and the energy efficiency of the demo LiVPO_4_F//Al_2_O_3_@Li_4_Ti_5_O_12_ open pouch cell with the 63 m WiSE and a constant charge capacity of 0.1 Ah at a rate of 0.2 C.

Based on the successfully inhibition of the ORR at the lithiated anode, a full open-cell was designed and fabricated. As the charge/discharge potentials of the LiMn_2_O_4_ cathode and the LiTi_2_(PO_4_)_3_ anode are within the electrochemical stability window of both the 28 m WiSE and the 1 m SiWE, the electrochemical performances of the LiMn_2_O_4_//Al_2_O_3_@LiTi_2_(PO_4_)_3_ open-cell in the 28 m WiSE and the LiMn_2_O_4_//LiTi_2_(PO_4_)_3_ open-cell in the 1 m SiWE were compared. The LiMn_2_O_4_//Al_2_O_3_@LiTi_2_(PO_4_)_3_ open-cell in the 28 m WiSE delivered a capacity of 62.4 Wh kg^−1^ (of total mass of the anode and the cathode) at a rate of 5 C (Fig. 5a) and displayed a high Coulombic efficiency of over 99.9% with a stable cycle life of >1000 cycles (Fig. 5b, c). In sharp contrast, the capacity of the LiMn_2_O_4_//LiTi_2_(PO_4_)_3_ open-cell in the 1 m SiWE decreased markedly due to the poor Coulombic efficiency (Supplementary Fig. 11, Fig. 5b, c). Self-discharge is a key issue for all batteries, Supplementary Fig. 12 and Supplementary Note 6 demonstrate that the fully charged LiMn_2_O_4_//LiTi_2_(PO_4_)_3_ open-cell with the 1 m SiWE was fully self-discharged by the O_2_ in the air after 10 h of rest under ambient conditions. The self-discharge performance of the LiMn_2_O_4_//Al_2_O_3_@LiTi_2_(PO_4_)_3_ open-cell in the 28 m WiSE was measured after resting for 10 h and the fully charged open-cell maintained 96.8% of the initial charged capacity (Supplementary Fig. 13). The capacity retention is still larger than 90% after 15 days of rest (Supplementary Fig. 14). Furthermore, the charging of the LiMn_2_O_4_//Al_2_O_3_@LiTi_2_(PO_4_)_3_ open cell with a constant capacity can minimize the effect of self-discharge on cycling stability. The constant capacity charge protocol enables the LiMn_2_O_4_//Al_2_O_3_@LiTi_2_(PO_4_)_3_ open-cell in the 28 m WiSE to maintain the same discharge capacity for 100 cycles at a low rate of 1 C, which results in the Coulombic efficiency of the LiMn_2_O_4_//Al_2_O_3_@LiTi_2_(PO_4_)_3_ open power cell at 1 C reaching >99.7% (Supplementary Fig. 13b). Even at a low rate of 0.2 C, the LiMn_2_O_4_//Al_2_O_3_@LiTi_2_(PO_4_)_3_ open cell shows a stable discharge capacity for 35 cycles under the constant charge capacity protocol (Supplementary Fig. 15).

To further enhance the cell energy density, a 2.7 V, 0.1 Ah LiVPO_4_F//Al_2_O_3_@Li_4_Ti_5_O_12_ open pouch demo cell was fabricated (Supplementary Fig. 16) using a high voltage LiVPO_4_F cathode and evaluated in the 63 m WiSE. The 63 m WiSE has a low O_2_ concentration (0.19 mg L^−1^) and wide electrochemical potential window of >3.15 V (1.76 V–4.91 V vs. Li^+^/Li) (Supplementary Fig. 17, Supplementary Note 7). The LiVPO_4_F//Al_2_O_3_@Li_4_Ti_5_O_12_ open pouch cell in the 63 m WiSE was cycled at a rate of 0.2 C and provided 170 Wh kg^−1^ (based on of the total mass of the anode and the cathode) (Supplementary Fig. 18, Supplementary Note 8 and Fig. 5d). The cell achieved over 50 cycles with a Coulombic efficiency of 96.8% (Fig. 5e).

Energy efficiency is another critical factor for new battery systems^28,29^. We have calculated the energy efficiency of the LiMn_2_O_4_//Al_2_O_3_@LiTi_2_(PO_4_)_3_ open-cell at the rate of 1 C and found it to be 90% (Supplementary Fig. 13C). The energy efficiency of the LiVPO_4_F//Al_2_O_3_@Li_4_Ti_5_O_12_ open pouch cell, cycled at 0.2 C, (Fig. 5e) is as high as 92.8%, which is comparable with the value (in the range of 86–98%) of commercial LIBs^29,30^. Therefore, the open-cell configuration developed here can satisfy the demand of the market.

To address the potential electrolyte leakage issue in the open-cell design, we added PVA (Poly(vinyl alcohol)) into the 28 m WiSE to form a gel electrolyte. The gel electrolyte can flow when the temperature is increased to 95 °C in order to facilitate battery assembly. When the electrolyte is cooled back to room temperature it forms a solid phase that can eliminate any electrolyte leakage. Supplementary Fig. 19 and Supplementary Note 9 show that the 28 m gel WiSE do not flow at room temperature. As shown in Supplementary Fig. 19b, c, the LiMn_2_O_4_//Al_2_O_3_@LiTi_2_(PO_4_)_3_ open pouch cell with the 28 m gel WiSE achieved similar electrochemical performance as the cell with the liquid 28 m WiSE. Supplementary Fig. 20, Supplementary Note 10 and Supplementary Movie 1 show that the LiMn_2_O_4_//Al_2_O_3_@LiTi_2_(PO_4_)_3_ open pouch cell with the 28 m gel WiSE was able to stably power a fan without any electrolyte leakage, even after the pouch cell was cut with scissors.

This aqueous open-cell configuration is a universal design that can also be used in other aqueous batteries. For example, the 35 m WiSE also effectively suppressed the ORR at the discharged electrode, which enabled the intercalated Na_3_Ti_2_(PO_4_)_3_ electrode in an open-cell configuration to maintain over 98.5% of the initial capacity after resting at open-circuit for 10 h (Supplementary Fig. 21). In addition, the 30 m WiSE also effectively suppressed the ORR at the Zn anode, as demonstrated by the much higher Coulombic efficiency (87.5%) for the Zn plating and stripping in the 30 m WiSE than in the 5 m ZnCl_2_ SiWE (53%) (Supplementary Fig. 22). The open-cell configuration has several other advantages: (1) some safety devices are not necessary within the cell such as an additional ventilation system; (2) they can be charged at a high voltage setting and high rate; and (3) they have a much lower up-front cost by removing the safety devices and sealing process.

## Discussion

The non-flammable aqueous batteries still carry the risk of explosion due to rapidly increasing internal pressure caused by side reaction gaseous products and thermal runaway. The WiSE can effectively suppress the electrolyte evaporation and the ORR reaction at the discharged anode. The ORR at the anode can be further suppressed by coating an Al_2_O_3_ nano-layer, which enables the batteries to operate in an open configuration. The LiMn_2_O_4_//Al_2_O_3_@LiTi_2_(PO_4_)_3_ open power cell achieves a stable cycle life of over 1000 cycles with a low self-discharge rate (90% capacity retention after 15 days). The 2.7 V LiVPO_4_F//Al_2_O_3_@Li_4_Ti_5_O_12_ open pouch cell achieves a high energy density of 170 Wh kg^−1^. The open configuration design provides enhanced resistance to thermal runaway and high-pressure explosion that can markedly improves the safety of LIBs.

## Methods

### Materials synthesis

LiMn_2_O_4_ and Li_4_Ti_5_O_12_ were purchased from MTI. LiVPO_4_F was supplied by ALeees. To synthesize the carbon-coated LiTi_2_(PO_4_)_3_. At first, 100 mL 2 wt% poly-vinyl-alcohol (PVA) aqueous solution was prepared, then Li_2_CO_3_, NH_4_H_2_PO_4_ and TiO_2_ were blended with it. A white solid product was formed when the water was evaporated after heating at 80 °C with continuous stirring. The product was heated at 900 °C for 10 h at a temperature increasing rate of 5 °C min^−1^ under the protection of N_2_ flow. During the heat-treatment process, it was in a porcelain boat, and the tube furnace was applied. Thermal decomposition vapor-deposition technology was applied to coat carbon on the surface of LiTi_2_(PO_4_)_3_. The as-prepared LiTi_2_(PO_4_)_3_ was placed in a tube furnace and further heated at 700 °C for 2 h, during which, the toluene vapor was carried by N_2_ through the tube. The flow rate of N_2_ is 1 L min^−1^. After that, the product was further heated at 900 °C for 2 h under the protection of N_2_ without toluene.

### Atomic layer deposition

The electrodes were coated with Al_2_O_3_ nano-layer using the atomic layer deposition (ALD) equipment (Beneq TFS 500). The carrier gas was high-purity N_2_ with a temperature of 150 °C. 20 precursor pulse cycles of ALD-Al_2_O_3_ were applied to form 2-nm-thickness layer on the surface of electrodes. During each cycle, alternating trimethylaluminum (4 s, Al precursor) and H_2_O (4 s, oxygen precursor) flows were separated by flows of N_2_ (4 s for the carrier gas, 10 s for the cleaning gas).

### Electrode preparation and electrochemical measurements

The working electrodes were prepared by mixing active materials (LiMn_2_O_4_, LiTi_2_(PO_4_)_3_, LiVPO_4_F, or Li_4_Ti_5_O_12_), conductive materials (carbon black, CB), and binder PTFE (polytetrafluoroethylene) in a weight ratio of 90:5:5 of active materials/CB/PTFE, then compressing them onto a stainless steel grid (LiMn_2_O_4_ and LiTi_2_(PO_4_)_3_), Ti grid (LiVPO_4_F), or Al grid (Li_4_Ti_5_O_12_) at 10 MPa. The linear sweep voltammetry (LSV) tests were performed using a three-electrodes cell, in which, the activated carbon was used as the counter electrode, the Ag/AgCl electrode was used as the reference electrode. The LSV measurements were carried out on a CHI660B electrochemical workstation. The galvanostatic charge/discharge tests of anodes were performed using a three-electrodes cell, in which, the activated carbon was used as the counter electrode, the Ag/AgCl electrode was used as the reference electrode. The galvanostatic charge/discharge of full cells were performed using the Arbin electrochemical working station.

The carbon black, Al_2_O_3_ (nano particles form Sigma) and carbon-coated LiTi_2_(PO_4_)_3_ inks were prepared as follows: (1) the active materials (5 mg) was dispersed in a mixture of isopropanol (2.5 mL) and Nafion solution (20 μL); (2) treat the mixture with ultra-sonication for 30 min. After that, the ink (10 μL) was deposited on a glassy carbon disk electrode (RDE or RRDE) and evaporated the solvent in air at room temperature. The electrochemical measurements were performed in a three-electrodes cell at ambient temperature. The RDE or RRDE was used as the working electrode, a platinum electrode as the counter electrode, and Ag/AgCl as the reference electrode. The data was recorded using a Gamry interface 1000.

### Materials characterizations

Raman measurements were carried out by a Horiba Jobin Yvon Labram Aramis using a 532 nm diode-pumped solid-state laser, attenuated to give ~900 μW power at the sample surface. Scanning electron microscopy (SEM) measurements were carried out by Hitachi SU-70 analytical SEM (Japan). Viscosity measurements were carried out using a CANNON-FENSKE viscometer. The surface chemistry of the electrodes after ALD coating was examined by XPS with a Kratos Axis 165 spectrometer. XPS data was collected using a monochromated Al Ka X-ray source (1486.7 eV). The working pressure of the chamber was lower than 6.6 × 10^−9^ Pa. All reported binding energy values were caLIBrated to the C 1*s* peak at 284.8 eV.

### Computational details

All density functional theory (DFT) calculations^31,32^ were performed using a Vienna Ab Initio Simulation Package (VASP)^33^ with projector augmented wave (PAW) method^34^. The exchange–correlation energy is described by the functional Perderw, Burke, and Ernzerhof (PBE) version of the generalized gradient approximation (GGA)^35^. And the energy cut-off for the plane wave basis is 520 eV. A vacuum layer of 12 Å was used for all calculated models. The energy of O_2_ is obtained from the Materials Project^36^. Visualization of the structures are made by VESTA^37^.

## Supplementary information

Supplementary Information

Description of Additional Supplementary Files

Supplementary Movie 1

## Data Availability

The data that support the plots within this paper and other findings of this study are available from the corresponding authors upon reasonable request.
