# Supplementary material for: Enabling safe aqueous lithium ion open batteries by suppressing oxygen reduction reaction
Source: Nat Commun. 2020 May 26;11:2638. doi: 10.1038/s41467-020-16460-w (PMC7250880; doi:10.1038/s41467-020-16460-w)
Supplement: Supplementary file 1 — Supplementary Information [file 41467_2020_16460_MOESM1_ESM.pdf]

Supplementary Information for

Enabling safe aqueous lithium ion open batteries by suppressing oxygen reduction reaction

Chen et al.

Supplementary Figures

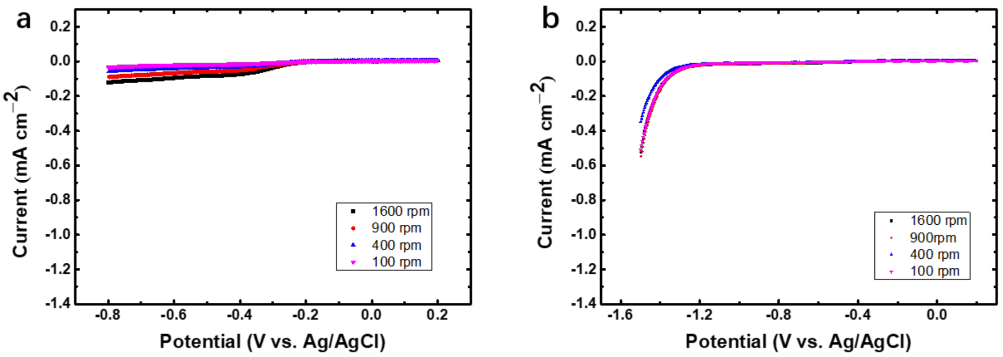

Supplementary Fig. 1 Linear sweep voltammetry (LSV) of carbon black in N_2-saturated electrolytes. (**a**) LSV of carbon black in N_2-saturated 1 m “Salt-in-water electrolytes” (SiWE) (1 m Li_2SO_4) at a scan rate of 10 mV s^{-1} at different RDE rotation rates; (**b**) LSV of carbon black in N_2-saturated 28 m “Water-in-salt electrolytes” (WiSE) (21 m LiTFSI + 7 m LiOTf) at a scan rate of 10 mV s^{-1} at different RDE rotation rates.

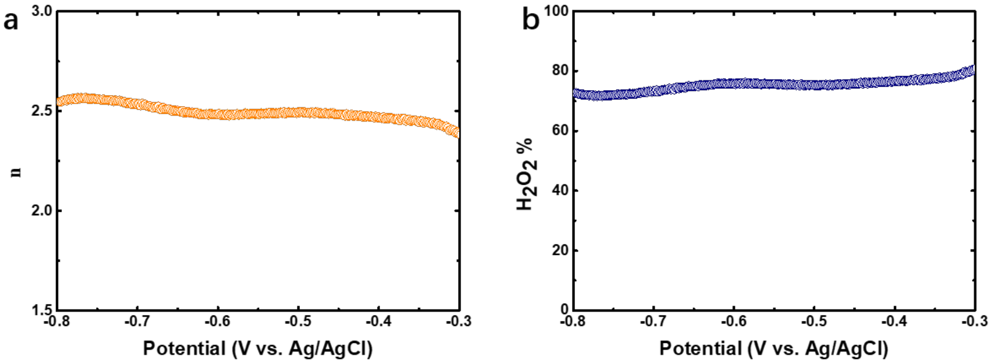

Supplementary Fig. 2 Overall electron transfer number of the ORR and corresponding percentage H_2O_2 in 1 m SiWE. (**a**) Plots of overall electron transfer number of the ORR in 1 m SiWE; (**b**) corresponding percentage of H_2O_2 produced as a function of electrode potential.

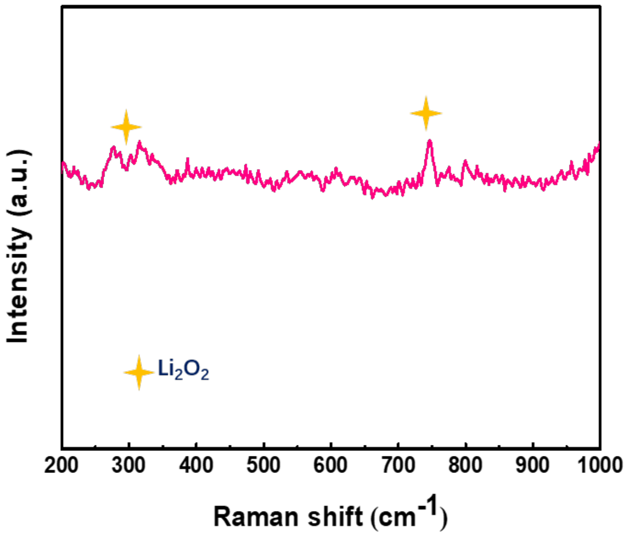

Supplementary Fig. 3 Raman spectrum of discharged carbon cathodes. The two distinguishing peaks (labeled by ✦) are from Li_2O_2.

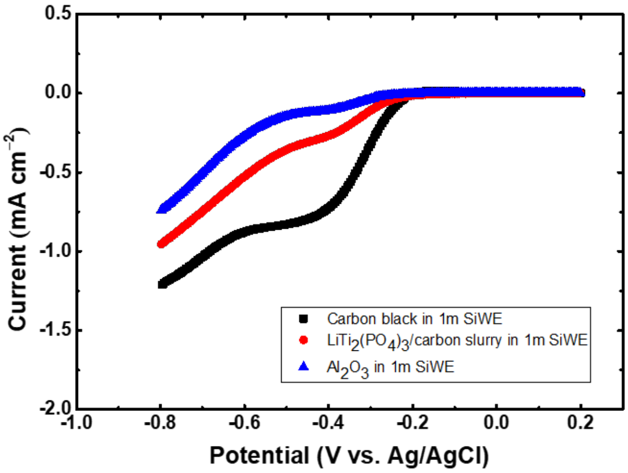

Supplementary Fig. 4 The ORR performance on Al_2O_3, $LiTi(PO_4)_3$/Carbon black and Carbon black in 1m SiWE. LSV of Al_2O_3, $LiTi(PO_4)_3$/Carbon black (90:5 by weight, the same ratio as in real electrode) and carbon black in O_2-saturated 1 m SiWE at a scan rate of 10 mV s^{-1} with a rotation rate of 1600 rpm. The loading on the RDE was 20 µg.

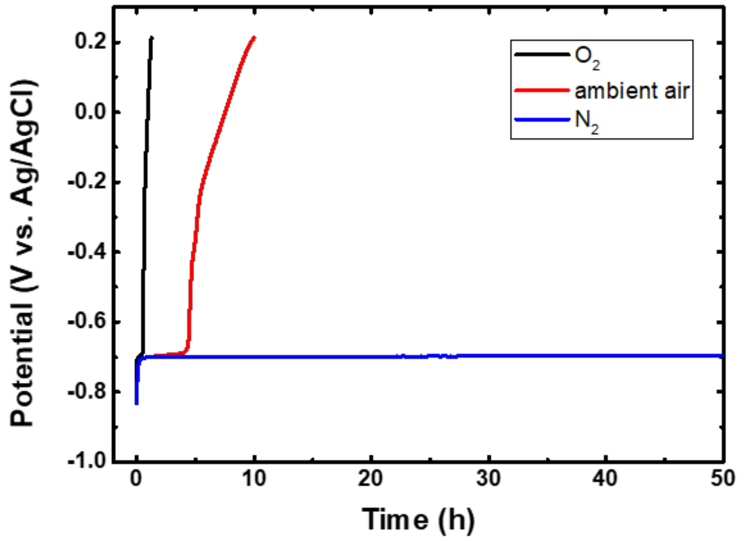

Supplementary Fig. 5 Effects of the ORR on the self-discharge performance of the lithiated $Li_3Ti_2(PO_4)_3$ electrode in a three-electrodes open-cell and the 1 m SiWE with pure O_2, ambient environment, and N_2 atmospheres. The loading of the $LiTi_2(PO_4)_3$ is 4 mg.

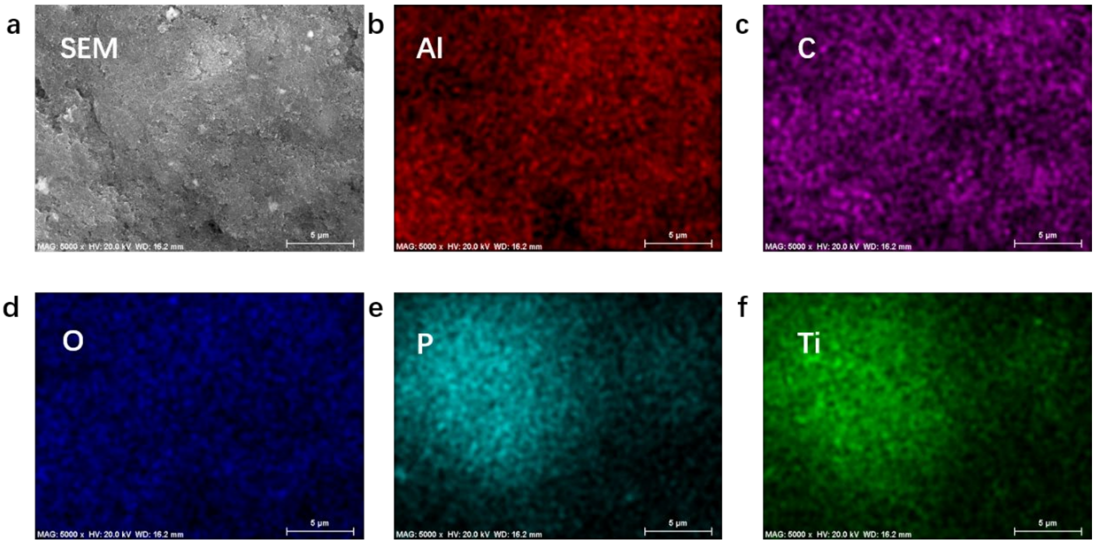

Supplementary Fig. 6 Elemental mapping of the ALD-Al_2O_3 coated $LiTi_2(PO_4)_3$ electrode. (**a**) SEM image; elemental mapping for (**b**) Al; (**c**) C; (**d**) O; (**e**) P; (**f**) Ti. The elemental mapping of the ALD-Al_2O_3 coated $LiTi_2(PO_4)_3$ electrode indicates that the Al_2O_3 is homogeneously coated on the electrode surface.

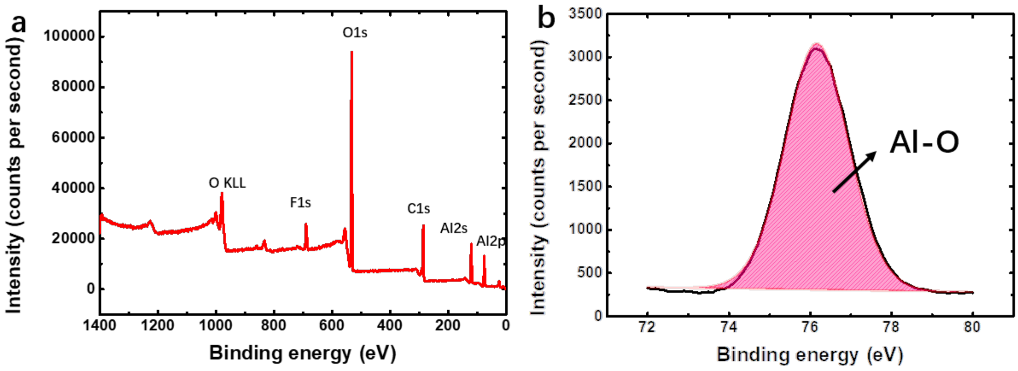

Supplementary Fig. 7 XPS analysis of the ALD-Al_2O_3 coated $LiTi_2(PO_4)_3$ electrode. (**a**) Full XPS spectrum of the ALD-Al_2O_3 coated $LiTi_2(PO_4)_3$ electrode; (**b**) XPS spectrum of the Al 2p peak for the ALD-Al_2O_3 coated $LiTi_2(PO_4)_3$ electrode.

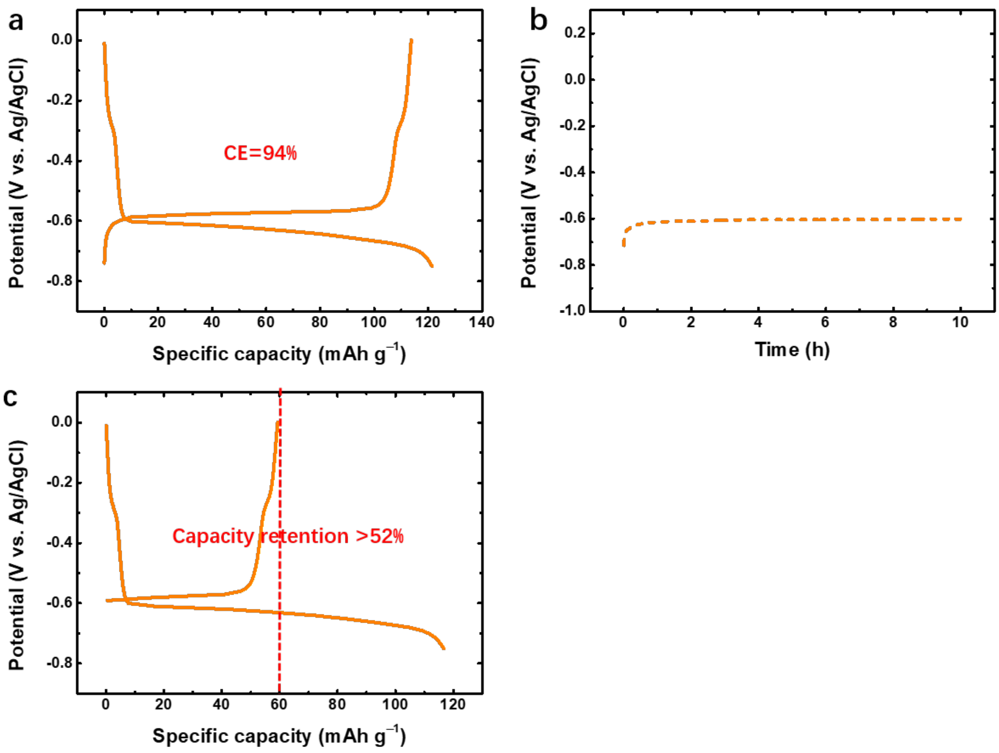

Supplementary Fig. 8 Effects of the ORR on the self-discharge of the lithiated Al_2O_3@$LiTi_2(PO_4)_3$ electrode in a three-electrodes open-cell with 10 m LiTFSI electrolyte. (**a**) The charge/discharge curves of the Al_2O_3@$LiTi_2(PO_4)_3$ electrode in the 10 m LiTFSI electrolyte at a current density of 0.5 A g^{-1}; (**b**) The open circuit potential profile of the lithiated Al_2O_3@$LiTi_2(PO_4)_3$ electrode in the 10 m LiTFSI electrolyte during a 10 h relaxation at open-circuit; (**c**) the lithiation potential profile and subsequent delithiation profile of the Al_2O_3@$LiTi_2(PO_4)_3$ electrode at a current density of 0.5 A g^{-1} in the 10 m LiTFSI electrolyte after a 10 h relaxation at open-circuit. These tests were conducted in an open-cell configuration exposed to the ambient atmosphere.

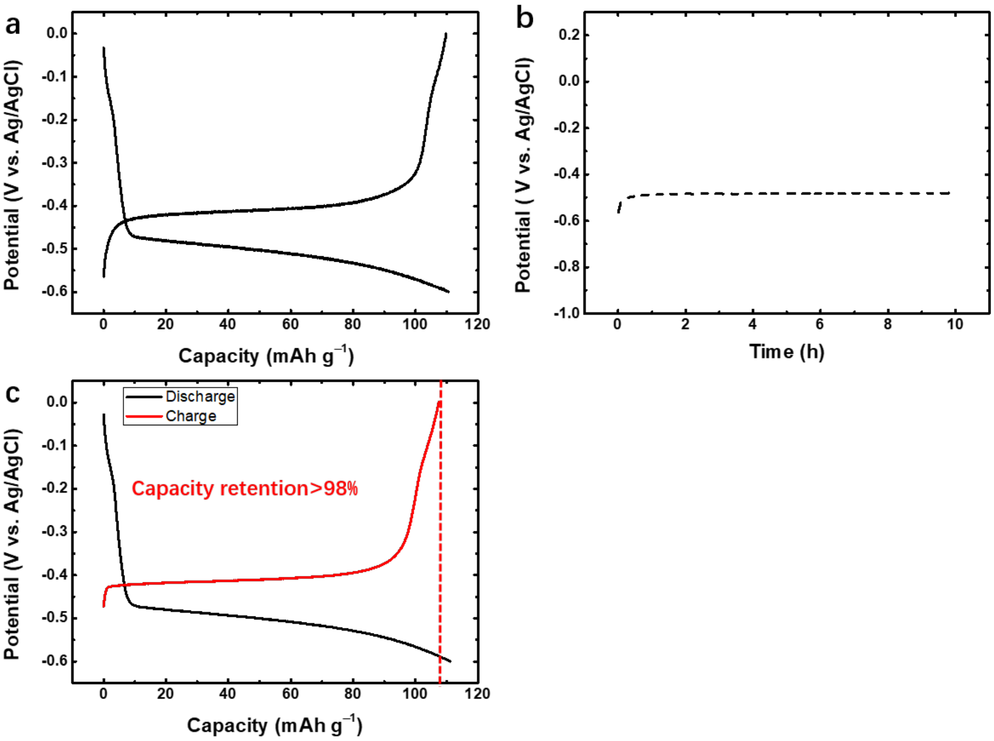

Supplementary Fig. 9 Effects of the ORR on the self-discharge of the lithiated $Li_3Ti_2(PO_4)_3$ electrode in a three-electrodes open-cell with the 30 m WiSE (28 m $ZnCl_2$ + 2 m LiCl). (**a**) The charge/discharge curves of the Al_2O_3@$LiTi_2(PO_4)_3$ electrode in the 30m WiSE at a current density of 0.1 A g^{-1}; (**b**) The open circuit potential curve of the fully lithiated Al_2O_3@$Li_3Ti_2(PO_4)_3$ electrode in the 30 m WiSE over 10 h; (**c**) The lithiation potential profile and the subsequent delithiation potential profile of the Al_2O_3@$LiTi_2(PO_4)_3$ electrode at a current density of 0.1 A g^{-1} after a 10 h rest in the 30 m WiSE. These tests were conducted in an open-cell configuration exposed to the ambient atmosphere.

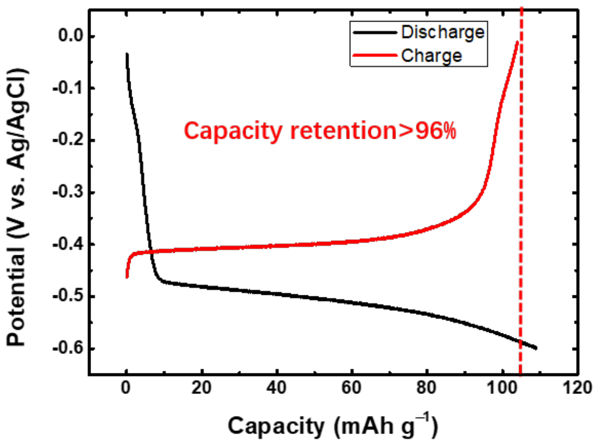

Supplementary Fig. 10 Effect of the ORR on the self-discharge of the lithiated $Li_3Ti_2(PO_4)_3$ electrode in a three-electrodes open-cell with the 30 m WiSE. The lithiation potential profile and the subsequent delithiation potential profile of the Al_2O_3@$LiTi_2(PO_4)_3$ electrode at a current density of 0.1 A g^{-1} after a 24 h rest in the 30 m WiSE exposed to the ambient environment.

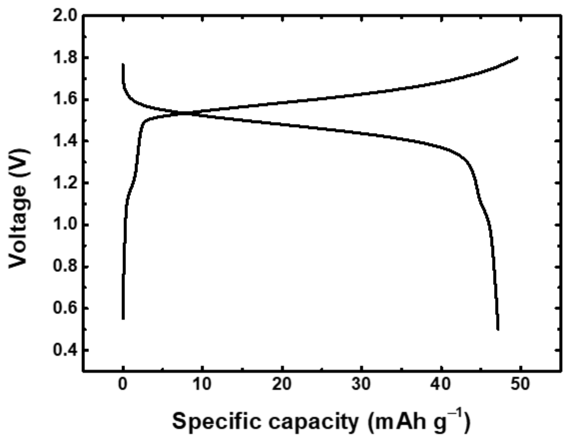

Supplementary Fig. 11 Typical charge/discharge curves of the $LiMn_2O_4$//$LiTi_2(PO_4)_3$ open-cell with the 1 m SiWE at a rate of 5 C.

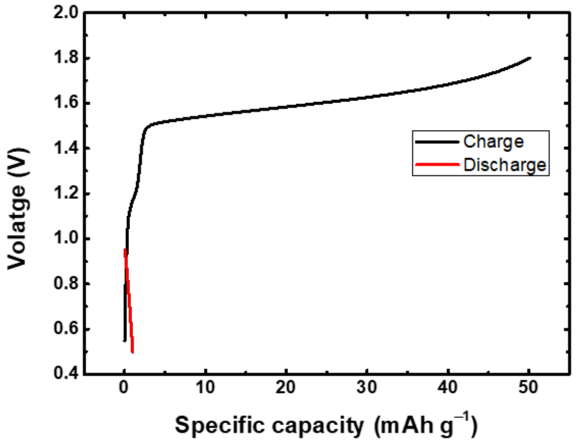

Supplementary Fig. 12 Self-discharge performance of the fully charged $LiMn_2O_4$//$LiTi_2(PO_4)_3$ open-cell with the 1 m SiWE exposed to the ambient environment. The $LiMn_2O_4$//$LiTi_2(PO_4)_3$ open-cell was charged at a rate of 1 C to the fully charged state. After a 10 h rest at open-circuit, the cell was discharged at 1 C. The charge and the discharge curves after 10 h rest are shown in the figure.

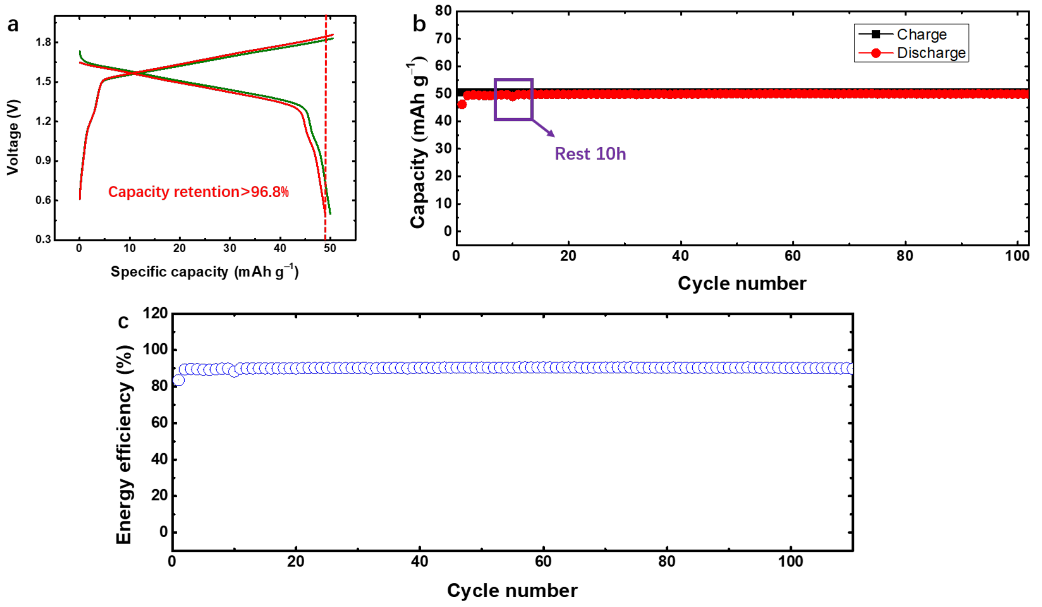

Supplementary Fig. 13 Electrochemical performance of the $LiMn_2O_4$//Al_2O_3@$LiTi_2(PO_4)_3$ open-cell with the 28 m WiSE and a constant charge capacity. (**a**) (green) The charge/discharge curves of the $LiMn_2O_4$//Al_2O_3@$LiTi_2(PO_4)_3$ open-cell with a constant charge capacity of 50.5 mAh g^{-1} at a rate of 1 C; (red) The charge curve of the $LiMn_2O_4$//Al_2O_3@$LiTi_2(PO_4)_3$ open-cell with a constant charge capacity of 50.5 mAh g^{-1} at a rate of 1 C and the subsequent discharge curve after a 10 h rest in an ambient environment at a rate of 1 C; (**b**) The cycling performance of the $LiMn_2O_4$//Al_2O_3@$LiTi_2(PO_4)_3$ open-cell with a constant charge capacity of 50.5 mAh g^{-1} at a rate of 1 C. The 10_{th} cycle included a 10 h rest in an ambient environment after charging. (**c**) The energy efficiency of the $LiMn_2O_4$//Al_2O_3@$LiTi_2(PO_4)_3$ open-cell at a rate of 1 C.

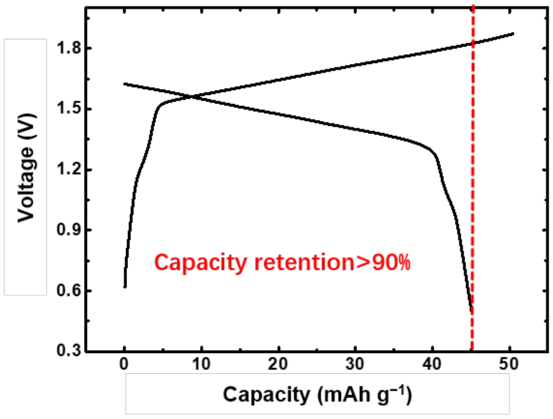

Supplementary Fig. 14 Self-discharge performance of the $LiMn_2O_4$//Al_2O_3@$LiTi_2(PO_4)_3$ open-cell with the 28 m WiSE. The charge curve of the $LiMn_2O_4$//Al_2O_3@$LiTi_2(PO_4)_3$ open-cell with a constant charge capacity of 50.5 mAh g^{-1} at a rate of 1 C and the subsequent discharge curve after a 15 day rest in an ambient environment at a rate of 1 C.

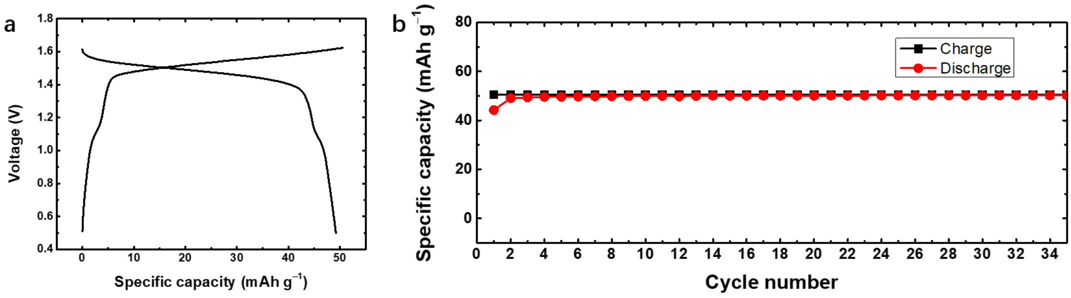

Supplementary Fig. 15 Electrochemical performance of the $LiMn_2O_4$//Al_2O_3@$LiTi_2(PO_4)_3$ open-cell in the 28 m WiSE with a constant charge capacity. (**a**) The charge/discharge curves of the $LiMn_2O_4$//Al_2O_3@$LiTi_2(PO_4)_3$ open-cell with a constant charge capacity of 50.5 mAh g^{-1} at a rate of 0.2 C; (**b**) The cycling performance of the $LiMn_2O_4$//Al_2O_3@$LiTi_2(PO_4)_3$ open-cell with a constant charge capacity of 50.5 mAh g^{-1} at a rate of 0.2 C.

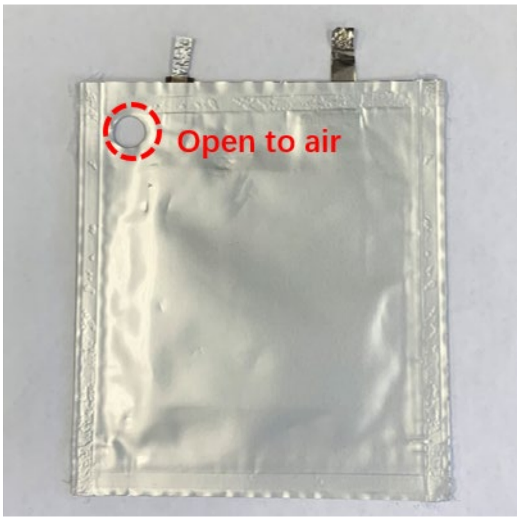

Supplementary Fig. 16 Picture of the demo $LiVPO_4F$//Al_2O_3@$Li_4Ti_5O_{12}$ open pouch cell with the 63 m WiSE (42 m LiTFSI + 21m Pyr_{13}·TFSI) (cell capacity is 0.1 Ah).

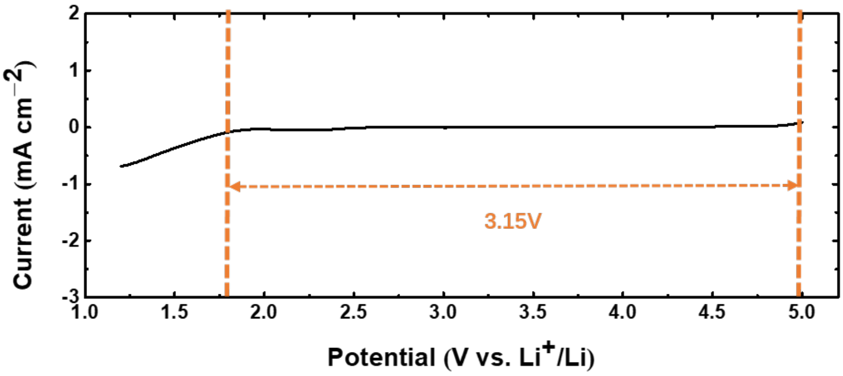

Supplementary Fig. 17 Electrochemical window of the 63 m WiSE. The LSV curves were measured on inactive current collectors (stainless steel foil) at a scanning rate of 5 mV s^{-1}.

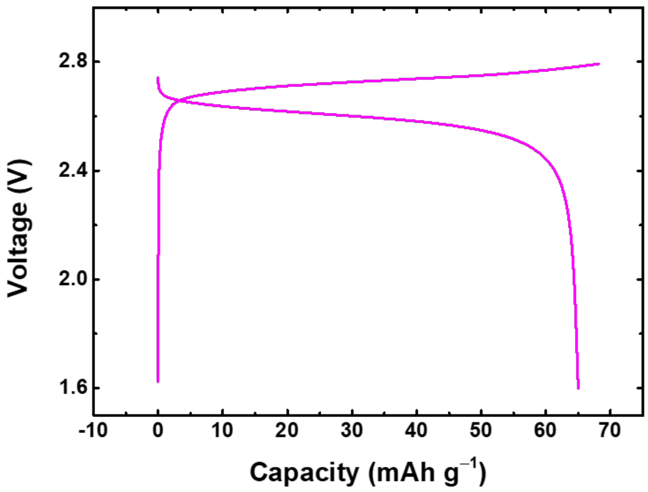

Supplementary Fig. 18 Electrochemical performance of the $LiVPO_4F$//Al_2O_3@$Li_4Ti_5O_{12}$ open-cell with the 63m WiSE and at a constant charge capacity.

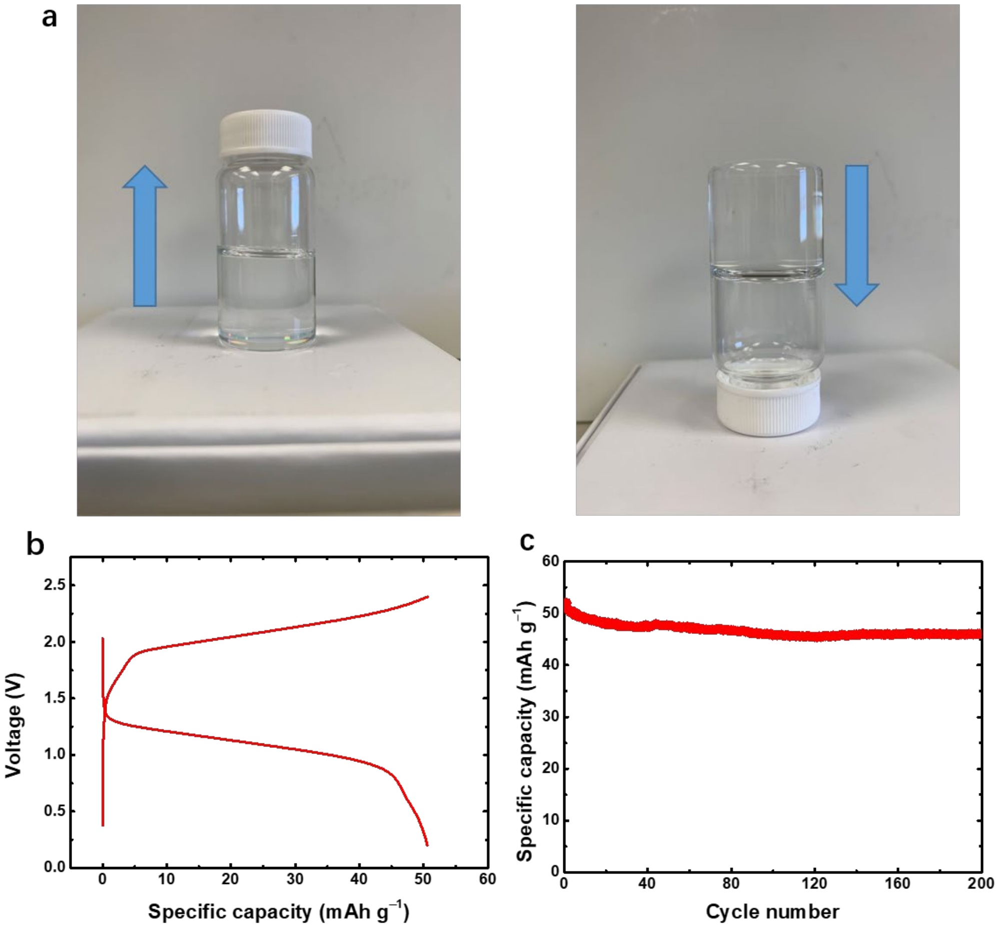

Supplementary Fig. 19 Picture and electrochemical performance of the 28 m gel WiSE (**a**) A picture of the 28m gel WiSE at room temperature and inverted to show that the electrolyte no longer flows; (**b**) The charge/discharge curves of the $LiMn_2O_4//Al_2O_3@LiTi_2(PO_4)_3$ open-cell with the 28 m gel WiSE at a rate of 5 C; (**c**) The cycling performance of the $LiMn_2O_4//Al_2O_3@LiTi_2(PO_4)_3$ open-cell with the 28 m gel WiSE at a rate of 5 C.

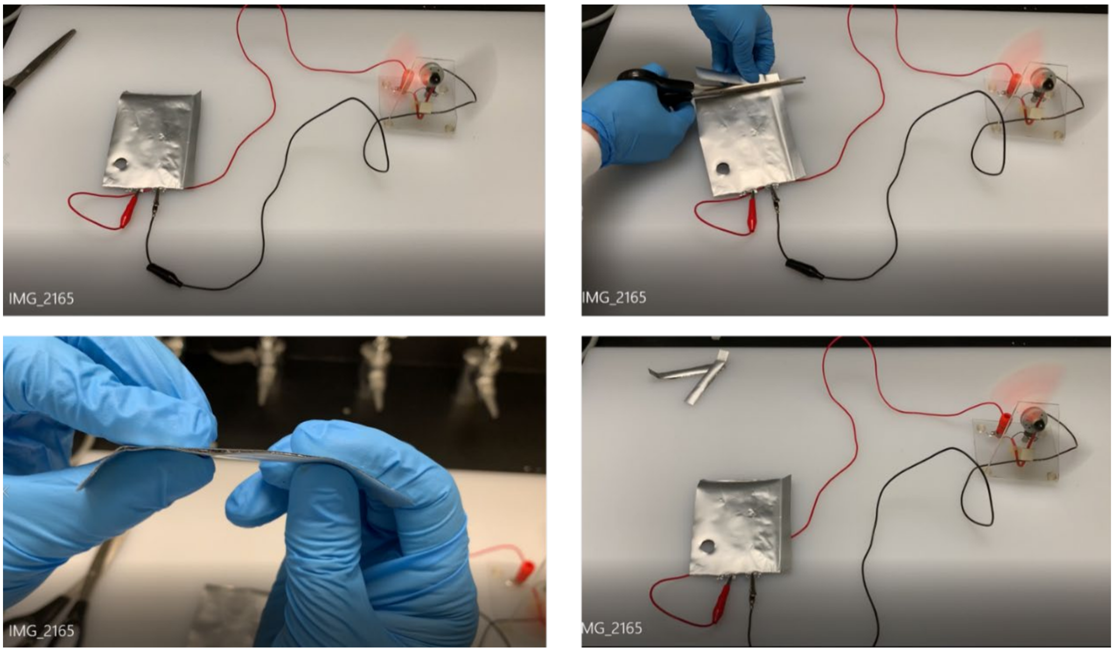

Supplementary Fig. 20 Picture of the $LiMn_2O_4$//Al_2O_3@$LiTi_2(PO_4)_3$ open pouch cell with the 28 m gel WiSE powering a fan.

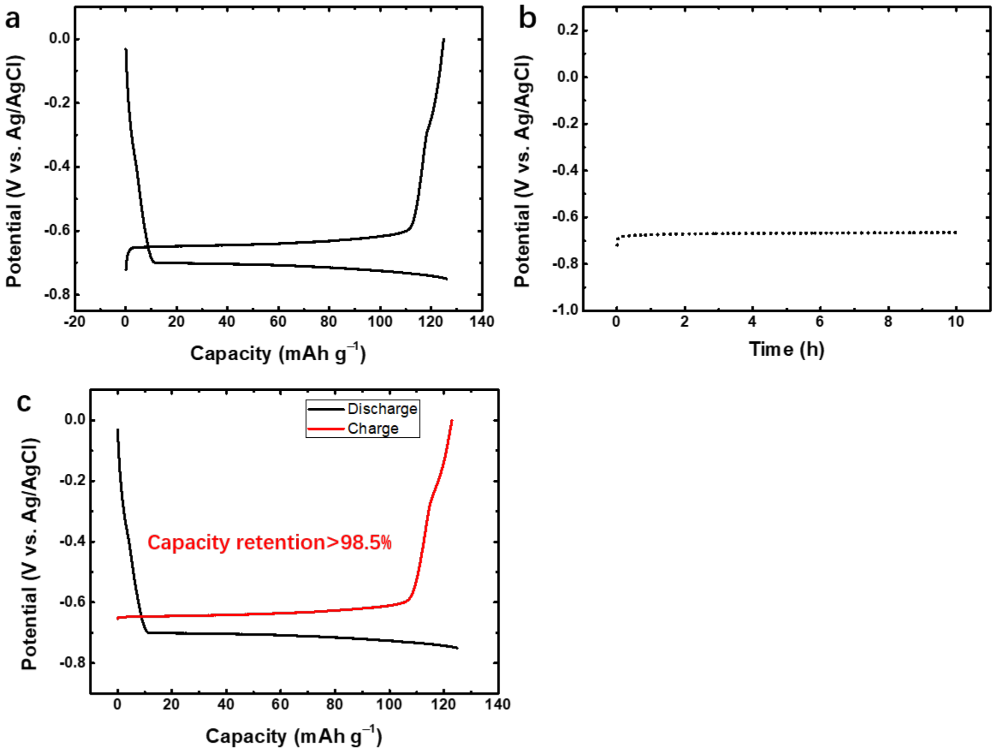

Supplementary Fig. 21 Effects of the ORR on the self-discharge of the sodiated $NaTi_2(PO_4)_3$ electrode in a three-electrodes open-cell. (**a**) The charge/discharge curves of the ALD-Al_2O_3 coated $NaTi_2(PO_4)_3$ electrode in the 35 m WiSE (35 m NaFSI-H_2O) at a current density of 0.1 A g^{-1}; (**b**) The open circuit potential profile of the sodiated Al_2O_3@$NaTi_2(PO_4)_3$ electrode in the 35 m WiSE during a 10 h relaxation at open-circuit; (**c**) The sodiation potential profile and subsequent desodiation profile of the Al_2O_3@$NaTi_2(PO_4)_3$ electrode at a current density of 0.1 A g^{-1} in the 35 m WiSE after a 10 h relaxation at open-circuit. These tests were conducted in an open-cell configuration with exposure to the ambient environment.

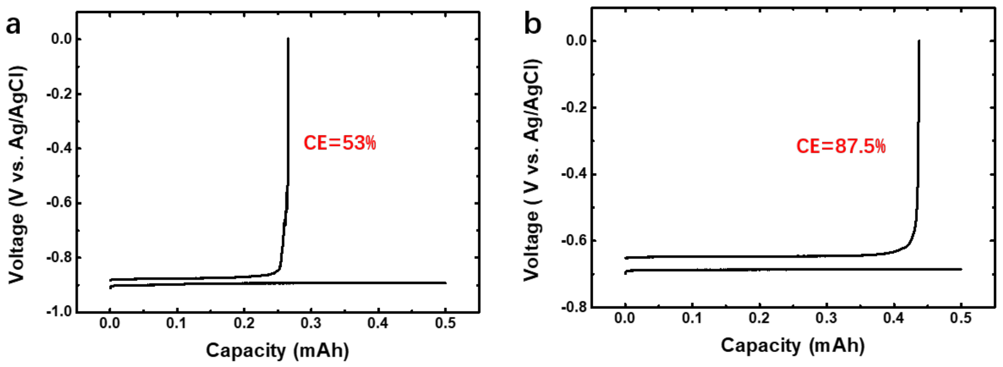

Supplementary Fig. 22 Effects of the ORR on the Coulombic efficiency of Zn plating/stripping. (**a**) The plating and stripping curves of Zn on the Ti foil in the 5 m SiWE (5 m $ZnCl_2$); (**b**) The plating and stripping curves of Zn on the Ti foil in the 30 m WiSE (30 m $ZnCl_2$). These tests were conducted in an open-cell configuration with exposure to the ambient environment.

Supplementary Notes

Supplementary Note 1. Overall electron transfer number of the ORR and corresponding percentage H_2O_2 in 1 m SiWE.

The amount of H_2O_2 was calculated based on the Supplementary Equation 1, where *jr* and *jd* are the ring and the disk current, respectively, and *N* is the collection efficiency (0.37).

$$H_2O_2\% = 2\frac{j_r}{N|j_d|+j_r} \times 100\% \quad (1)$$

Supplementary Note 2. Raman spectrum of discharged carbon cathodes.

The carbon electrode was discharged with a constant current of −0.1 mA for 20 h in the 28 m WiSE. Before the Raman characterization, the carbon cathode was rinsed by DME (Dimethoxyethane) several times.

Supplementary Note 3. The ORR performance on Al_2O_3, $LiTi(PO_4)_3$/Carbon black and Carbon black in 1m SiWE.

Supplementary Fig. 4 compares the RDE curves of Al_2O_3, $LiTi_2(PO_4)_3$/Carbon black (90:5 in weight, the same ratio as in real electrode) and Carbon black with 1 m SiWE. The ORR kinetics at the $LiTi_2(PO_4)_3$/Carbon black surface are faster than at the Al_2O_3 surface, but it is slower than at the carbon black surface. The limiting current density at 1600 rpm at the $LiTi(PO_4)_3$/Carbon black surface was 0.31 mA cm^{-2}, which is larger than at the Al_2O_3 surface (0.107 mA cm^{-2}) and lower than at the carbon black surface (0.85 mA cm^{-2}). Therefore, the Al_2O_3 nano layer coating would significantly slow down the ORR kinetics at the $LiTi_2(PO_4)_3$/Carbon black electrode surface.

Supplementary Note 4. Effects of the ORR on the self-discharge performance of the lithiated $Li_3Ti_2(PO_4)_3$ electrode in different atmospheres.

As shown in Supplementary Fig. 5, the open circuit potential of the fully lithiated $Li_3Ti_2(PO_4)_3$ electrode in the 1 m SiWE after exposure to the ambient environment could be sustained at the equilibrium potential only for about 4 h before the potential quickly rose to +0.2V after 10 h, suggesting that the fully lithiated $Li_3Ti_2(PO_4)_3$ electrode was fully self-discharged after 10 h in the ambient environment. When the fully lithiated $Li_3Ti_2(PO_4)_3$ electrode in the 1 m SiWE was introduced with pure O_2, it was completely self-discharged after only 0.5 h. However, in the absence of O_2 (in a N_2 atmosphere), it can maintain its potential for 50 h. These results clearly demonstrate that even in an ambient environment (not pure O_2), the O_2 in the air can quickly chemically oxidize the lithiated anodes in dilute aqueous electrolytes, resulting in fast self-discharge.

Supplementary Note 5. Effects of the ORR on the self-discharge of the lithiated Al_2O_3@$LiTi_2(PO_4)_3$ electrode in 10 m LiTFSI electrolyte.

The Coulombic efficiency of the ALD-Al_2O_3 coated $LiTi_2(PO_4)_3$ in the 10 m LiTFSI electrolyte at a current density of 0.5 A g^{-1} is 94% (Supplementary Fig. 8a), which is higher than the 89% Coulombic efficiency in the 1 m SiWE, but lower than the 99.9% Coulombic efficiency in the 28 m WiSE. The capacity retention of the fully lithiated $Li_3Ti_2(PO_4)_3$ electrode after opening it to the air for 10 h is ~52% (Supplementary Fig. 8b). All of these results indicate that the 10 m LiTFSI electrolyte can partially suppress the ORR. The solubility of O_2 in the 10 m LiTFSI electrolyte is 1.52 mg L^{-1}, which is lower than 1.97 mg L^{-1} in the 1 m SiWE, but it is much higher than in the 28m WiSE (0.95 mg L^{-1}). The relatively low O_2 solubility reduces the ORR kinetics to a certain degree; however, the 10 m LiTFSI electrolyte cannot form a passivation layer on the anode to further reduce the ORR kinetics. That is another reason for the relatively poor performance of the lithiated $Li_3Ti_2(PO_4)_3$ in the 10 m LiTFSI electrolyte when compared to the performance in the 28 m WiSE.

Supplementary Note 6. Self-discharge performance of the fully charged $LiMn_2O_4$//$LiTi_2(PO_4)_3$ open-cell with the 1 m SiWE exposed to the ambient environment.

We fabricated a $LiMn_2O_4$//$LiTi_2(PO_4)_3$ open full cell with the 1 m SiWE. The full cell was fully charged and then allowed to rest at open-circuit for 10 h under an ambient environment. As demonstrated in Supplementary Fig. 12, almost all of the charge capacity was self-discharged after 10 h of open-circuit resting due to the ORR on the lithiated anode. This is consistent with the results in Supplementary Fig. 5.

Supplementary Note 7. Electrochemical window of the 63 m WiSE.

The electrochemical stability window of the 63 m WiSE was evaluated with LSV on inactive stainless-steel foil electrodes. Supplementary Fig. 17 indicates that the potential for hydrogen and oxygen evolution are 1.76 V and 4.91 V, respectively.

Supplementary Note 8. Electrochemical performance of the $LiVPO_4F$//Al_2O_3@$Li_4Ti_5O_{12}$ open-cell with the 63m WiSE.

The charge/discharge curves of the $LiVPO_4F$//Al_2O_3@$Li_4Ti_5O_{12}$ open-cell with the 63 m WiSE at a constant charge capacity of 68 mAh g^{-1} and at a rate of 0.2 C. It shows that, this open-cell can deliver a specific discharge capacity of 65 mAh g^{-1} with a small over-potential.

Supplementary Note 9. Picture and electrochemical performance of the 28 m gel WiSE.

Supplementary Fig. 19a shows that the 28 m gel WiSE does not flow at room temperature. As shown in Supplementary Fig. 19b and 19c, the $LiMn_2O_4$//Al_2O_3@$LiTi_2(PO_4)_3$ open-cell with the 28 m gel WiSE delivered a comparable charge/discharge behavior and cycling stability to the $LiMn_2O_4$//Al_2O_3@$LiTi_2(PO_4)_3$ open-cell with the 28 m liquid WiSE. In fact, the LiTFSI concentration in the PVA-H_2O system can reach 35 m, which can further enhance the electrolyte's electrochemical stability window, reduce the oxygen solubility and the ORR rate, and suppress the electrolyte's solvent evaporation.

Supplementary Note 10. Picture of the $LiMn_2O_4$//Al_2O_3@$LiTi_2(PO_4)_3$ open pouch cell with the 28 m gel WiSE powering a fan.

Supplementary Fig. 20 shows that the $LiMn_2O_4$//Al_2O_3@$LiTi_2(PO_4)_3$ open pouch cell with the 28 m gel WiSE can stably power a fan without any electrolyte leakage. Even after the pouch cell was cut with scissors, it could still power the fan stably without electrolyte leakage from the cell. Supplementary Movie 1 in supporting information demonstrates that the cell powers the fan during cutting.
