# Supplementary material for: Enabling safe aqueous lithium ion open batteries by suppressing oxygen reduction reaction
Source: Nat Commun. 2020 May 26;11:2638. doi: 10.1038/s41467-020-16460-w (PMC7250880; doi:10.1038/s41467-020-16460-w)
Supplement: Supplementary file 2 — Description of Additional Supplementary Files [file 41467_2020_16460_MOESM2_ESM.pdf]

Description of Additional Supplementary Files

Supplementary Movie 1 | the open cell powers a fan stably during cutting.
